# Hepatitis Flares or Hepatic Decompensation after Discontinuation of Tenofovir Disoproxil Fumarate and Entecavir in Non-Cirrhotic Hepatitis B e Antigen-Negative Patients

**DOI:** 10.3390/jcm12247565

**Published:** 2023-12-08

**Authors:** Yi-Jie Huang, Tsai-Chung Li, Cheng-Hsu Chen, Chung-Hsin Chang, Szu-Chia Liao, Shou-Wu Lee, Yen-Chun Peng, Teng-Yu Lee, Jun-Sing Wang

**Affiliations:** 1 Department of Public Health, College of Public Health, China Medical University, Taichung 406040, Taiwan; jessiehij@hotmail.com (Y.-J.H.); tcli@mail.cmu.edu.tw (T.-C.L.); 2 Department of Medicine, School of Medicine, National Yang Ming Chiao Tung University, Taipei 112304, Taiwan; pychunppp@gmail.com; 3 Division of Gastroenterology and Hepatology, Department of Internal Medicine, Taichung Veterans General Hospital, Taichung 407219, Taiwan; b8501122@tmu.edu.tw (C.-H.C.); scarlett@vghtc.gov.tw (S.-C.L.); ericest@vghtc.gov.tw (S.-W.L.); tylee@vghtc.gov.tw (T.-Y.L.); 4 Department of Healthcare Administration, College of Medical and Health Science, Asia University, Taichung 413305, Taiwan; 5 Division of Nephrology, Department of Internal Medicine, Taichung Veterans General Hospital, Taichung 407219, Taiwan; cschen920@gmail.com; 6 Department of Post-Baccalaureate Medicine, College of Medicine, National Chung Hsing University, Taichung 402202, Taiwan; 7 Division of Endocrinology and Metabolism, Department of Internal Medicine, Taichung Veterans General Hospital, Taichung 407219, Taiwan

**Keywords:** chronic hepatitis B, hepatitis flares, hepatic decompensation

## Abstract

Hepatic events can occur after discontinuing antiviral therapy. We investigated factors associated with hepatitis flares and hepatic decompensation after discontinuing tenofovir disoproxil fumarate (TDF) and entecavir (ETV). Hepatitis flares within 6 months and hepatic decompensation were compared between non-cirrhotic hepatitis B e antigen-negative patients after discontinuing TDF or ETV by using the Cox proportional hazard model. The cumulative rates of hepatitis flare at 6 months after discontinuing ETV and TDF were 2% and 19%, respectively (*p* < 0.001). The respective rates of hepatic decompensation at 6 months were 0% and 7% (*p* = 0.009). Higher alanine aminotransferase (ALT) (AASLD criteria) at the end of treatment (EOT) (HR = 4.93; *p* = 0.001), an off-therapy dynamic change in HBV DNA (rapid rebound of HBV DNA from the nadir, ≥1 log_10_ IU/mL per month) (HR = 10.7; *p* < 0.001), and the discontinuation of TDF (HR = 6.44; *p* = 0.006) were independently associated with hepatitis flares within 6 months. Older age (HR = 1.06; *p* < 0.001) and an off-therapy dynamic change in HBV DNA (HR = 3.26; *p* = 0.028) were independently associated with hepatic decompensation after the discontinuation of antiviral therapy. In summary, we demonstrated several factors associated with hepatitis flares and hepatic decompensation after discontinuing antiviral therapy in non-cirrhotic hepatitis B e antigen-negative patients.

## 1. Introduction

Chronic hepatitis B (CHB) infection can cause hepatic decompensation, cirrhosis, and hepatocellular carcinoma (HCC), which caused 820,000 deaths in 2019 [1,2,3]. Hepatitis B surface antigen (HBsAg) seroclearance, called functional cure, is the most important treatment end point and can reduce the risk of HCC [4]. However, the cumulative rate of HBsAg loss is low, and Chevaliez S et al. [5] reported that an estimated 52.2 years is needed to achieve HBsAg seroclearance under long-term antiviral therapy. Therefore, infinite therapy is needed to eradicate hepatitis B virus for the majority of CHB patients treated with antiviral drugs. 

There are several concerns with respect to long-term antiviral therapy, including financial burden, drug compliance, and patients’ willingness. Recent studies in Asian countries found higher HBsAg loss rates in CHB patients after the withdrawal of antiviral therapy compared to those who continued [6]. Asian and European liver associations have provided guidance on stopping antiviral therapy for non-cirrhotic hepatitis B e antigen (HBeAg)-negative patients after receiving an 18-month duration of treatment consolidation [7,8]. However, previous studies demonstrated that the 12-month virological relapse rate and biochemical relapse rate were up to 76% and 42% in patients who discontinued tenofovir disoproxil fumarate (TDF), and 69% and 29% in patients who discontinued entecavir (ETV), respectively [9]. Hirode G et al. [10] reported that the 12-month cumulative rate of hepatic decompensation in HBeAg-negative patients after treatment withdrawal was 1%.

Hence, the best strategy for off-therapy surveillance should be established to avoid further liver deterioration. This study aimed to investigate the impact of an off-therapy dynamic change in HBV DNA levels in non-cirrhotic HBeAg-negative patients and to determine whether other factors at end of treatment (EOT) could help to predict sequential hepatitis flares or hepatic decompensation after the discontinuation of antiviral therapy.

## 2. Materials and Methods

### 2.1. Patients

This was a retrospective cohort study conducted at Taichung Veteran General Hospital. Non-cirrhotic HBeAg-negative patients who discontinued ETV or TDF after achieving HBV DNA undetectability on 3 occasions, each at least 6 months apart, were enrolled consecutively between July 2010 and May 2022. Patients were excluded if there was evidence of any of the following: (1) age younger than 18 years old, (2) treatment concomitant with chemotherapy or immunosuppressant agents during the previous antiviral therapy or after the discontinuation of antiviral therapy, (3) current coinfection with the hepatitis C virus or human immunodeficiency virus, and (4) history of HCC or other malignancy. The diagnosis of cirrhosis was based on the characteristic findings on abdominal ultrasonography including the appearance of a nodular surface and splenomegaly. 

The study was approved by the Institutional Review Board of our institution (Taichung Veterans General Hospital IRB: CE-18232A). Informed consent was not required as this was a retrospective study. 

### 2.2. Assessment and Follow-Up Evaluation

The baseline data were defined as the date of EOT. Patients were assessed using laboratory tests that included a liver biochemical test and serum HBV DNA at EOT and every 3–6 months after the discontinuation of antiviral therapy. If serum alanine aminotransferase (ALT) elevation was detected, additional serum HBV DNA would be measured. The definition of ALT normalization was a value within the upper limit of normal range (ULN) and was set at 50 U/L for men and 35 U/L for women as the commonly used reference intervals in this study. Another cut-off ALT value recommended by the American Association for the Study of Liver Diseases (AASLD) was set at 35 U/L for men and 25 U/L for women [11]. The aspartate aminotransferase to platelet ratio index (APRI) and Fibrosis-4 (FIB-4) score were calculated at EOT according to the following equations: (AST [U/L] × 100)/(ULN_AST_ × platelet count [10^9^/L]) and Age (years) × AST (U/L)/platelet count (×10^9^/L) × √ALT (U/L), respectively. The consolidation period was defined as the duration of therapy between patients’ first instance of HBV DNA being undetectable and patients discontinuing antiviral therapy. Patients after discontinuing antiviral therapy were followed in the same medical center and censored at the occurrence of the first episode of hepatitis flares (in the primary analysis) and hepatic decompensation (in the secondary analysis), loss to follow-up, or when they received antiviral therapy (whichever came first). 

### 2.3. Definition of Off-Therapy Response

The primary end point was the occurrence of hepatitis flares within 6 months after discontinuing TDF or ETV. A hepatitis flare was defined as ALT elevation to a level higher than 5 times the ULN with virological relapse (defined as HBV DNA > 2000 IU/mL) [8,12]. The secondary end point was the occurrence of hepatic decompensation with virological relapse after discontinuing TDF or ETV. Hepatic decompensation was defined as the presentation of several symptoms: jaundice with a total serum bilirubin level ≥2 mg/dL, the prolongation of prothrombin time (PT) ≥3 s, and/or the development of ascites/encephalopathy [13]. 

### 2.4. Laboratory Methods

HBV DNA was determined by a real-time PCR assay with Roche CobasTaqMan HBV Test (Roche Diagnostics, Branchburg, NJ, USA). HBsAg and HBeAg were determined by an electrochemiluminescence immunoassay (Roche Diagnostics, Mannheim, Germany). The serum quantitative HBsAg level (qHBsAg) was determined by a Roche Elecsys HBsAg II quant assay, which has a range of 0.05 IU/mL to 52,000 IU/mL (Abbott Diagnostics, Sligo, Ireland).

### 2.5. Statistical Analysis

Statistical tests were performed using SPSS Statistics for Windows, version 22.0 (IBM Corp., Armonk, NY, USA). Categorical variables were presented as the number of cases (percentage) and compared by the Chi-squared test. Continuous variables were expressed as the median ± interquartile range (IQR) and compared by the Mann–Whitney U test. The cumulative rates of hepatitis flare and hepatic decompensation after discontinuing TDF and ETV were calculated using the Kaplan–Meier method and compared with the log-rank test between patients after discontinuing TDF and ETV. The Cox proportional hazard model was used to analyze factors associated with hepatitis flares within 6 months and hepatic decompensation. Significant factors (*p* < 0.05) in the univariate analysis were subjected to multivariate analysis to determine independent predictive factors. Subgroup analysis was conducted to explore the associations of a treatment cessation regimen with the occurrence of hepatitis flares within 6 months and hepatic decompensation after discontinuing TDF or ETV. 

## 3. Results

### 3.1. Patients’ Characteristics at EOT and Follow-Up 

A total of 369 non-cirrhotic HBeAg-negative CHB patients who discontinued TDF and ETV were included in this study. At EOT, the clinical characteristics and demographics of these patients were not significantly different, with the exception of age. Patients who discontinued TDF were younger than those discontinuing ETV (50.60 vs. 54.43 years, *p* = 0.037). Patients who discontinued TDF and ETV had a similar duration of antiviral therapy and consolidation (Table 1). The median follow-up duration was 22.99 (IQR 12.07–49.52) months after EOT.

### 3.2. Factors Associated with Hepatitis Flares within 6 Months

**Figure 1:**
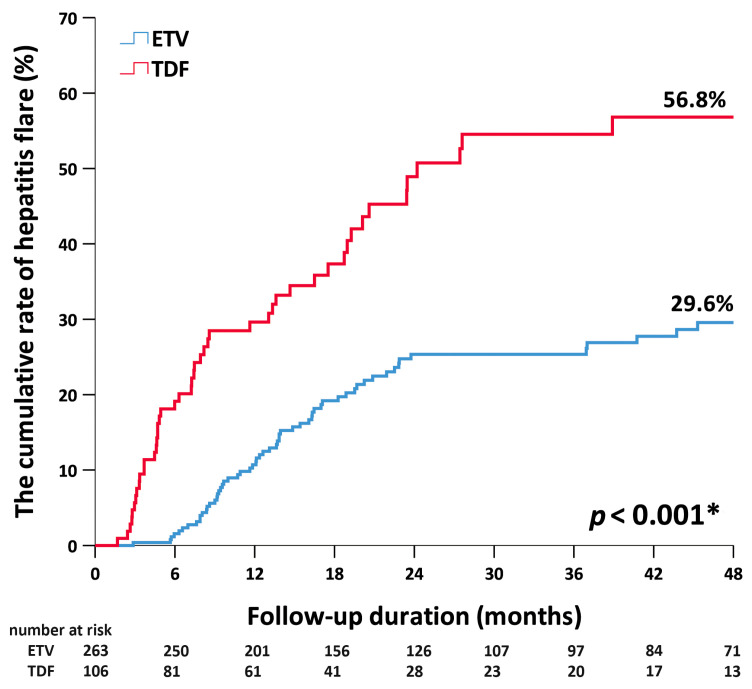
Cumulative rates of hepatitis flare by antiviral therapy. ETV, entecavir. TDF, tenofovir disoproxil fumarate. * < 0.05.

Overall, hepatitis flares within 6 months were observed in 24 patients, 20 and 4 of whom had discontinued TDF and ETV, respectively. Figure 1 shows the cumulative rates of hepatitis flare at 6 months in patients after the discontinuation of ETV and TDF were 2% and 19%, respectively. The respective rates at 1 year were 10% and 30% (*p* < 0.001). Multivariate Cox regression showed that an ALT that was higher than the AASLD criteria threshold at EOT (hazard ratio (HR) = 4.93; 95%CI, 1.84–13.18; *p* = 0.001), an off-therapy dynamic change in HBV DNA (HR = 10.7; 95%CI, 3.3–34.68; *p* < 0.001), and the discontinuation of TDF (HR = 6.44; 95%CI, 1.7–24.01; *p* = 0.006) were independent predictors for hepatitis flares within 6 months after the discontinuation of antiviral therapy (Table 2). 

**Figure 2:**
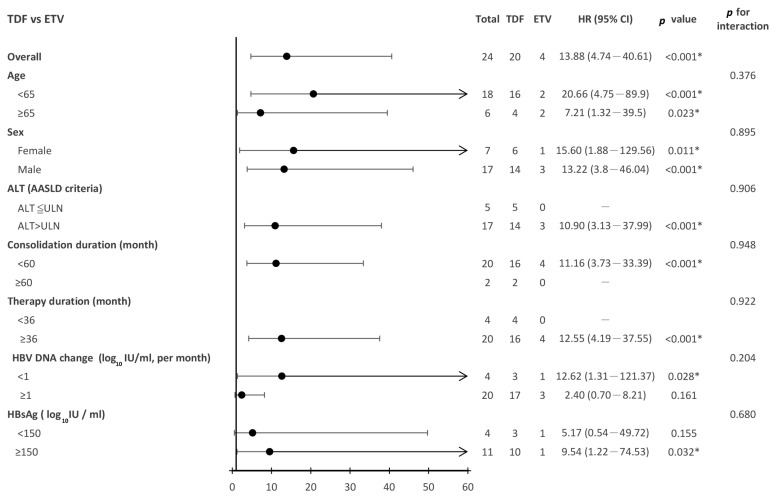
Subgroup analysis for hepatitis flares for patients discontinuing TDF vs. ETV. TDF, tenofovir disoproxil fumarate. ETV, entecavir. * < 0.05.

The hazard ratios for hepatitis flares within 6 months for patients after the discontinuation of TDF versus ETV across the different patient subgroups are shown in Figure 2. The risk of hepatitis flares was significantly higher in patients after the discontinuation of TDF than in those discontinuing ETV (HR = 13.88; 95%CI 4.74–40.61; *p* < 0.001). A higher risk of hepatitis flares in patients after the discontinuation of TDF therapy was consistently observed in various subgroups, including patients with an off-therapy HBV DNA change less than 1 log_10_ IU per month, qHBsAg at EOT ≥ 150 IU/mL, consolidation therapy less than 60 months, previous antiviral therapy more than 36 months, and an ALT higher than the AASLD criteria threshold, regardless of age (<65 or ≥65) or sex. 

### 3.3. Factors Associated with Hepatic Decompensation

**Figure 3:**
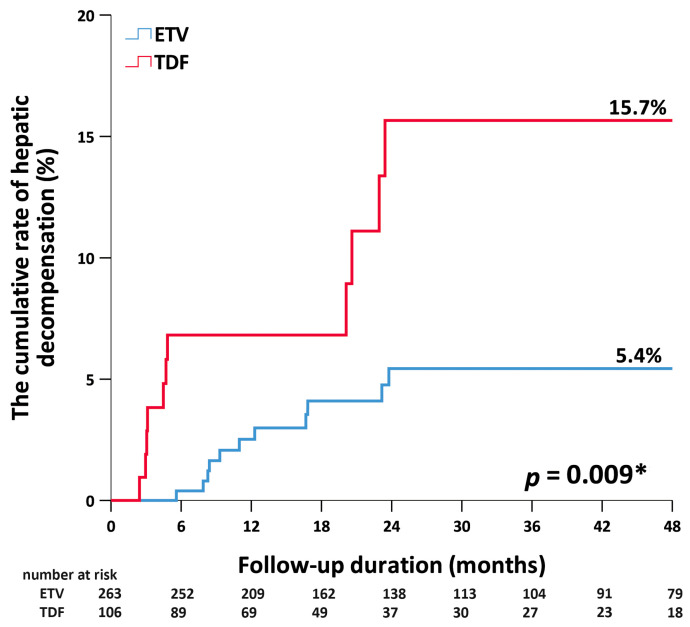
Cumulative rates of hepatic decompensation by antiviral therapy. ETV, entecavir. TDF, tenofovir disoproxil fumarate. * < 0.05.

Overall, hepatic decompensation was observed in 23 patients, 11 and 12 of whom discontinued TDF and ETV, respectively. Figure 3 shows that the 0.5-year cumulative rates of hepatic decompensation in patients after the discontinuation of ETV and TDF were 0% and 7%, respectively. The respective rates at 1 year were 3% and 8% (*p* = 0.009). Multivariate Cox regression showed that older age (HR = 1.06; 95%CI 1.03–1.1; *p* < 0.001) and an off-therapy dynamic change in HBV DNA (HR = 3.26; 95%CI 1.14–9.33; *p* = 0.028) were independent predictors for hepatic decompensation after the discontinuation of antiviral therapy (Table 3).

**Figure 4:**
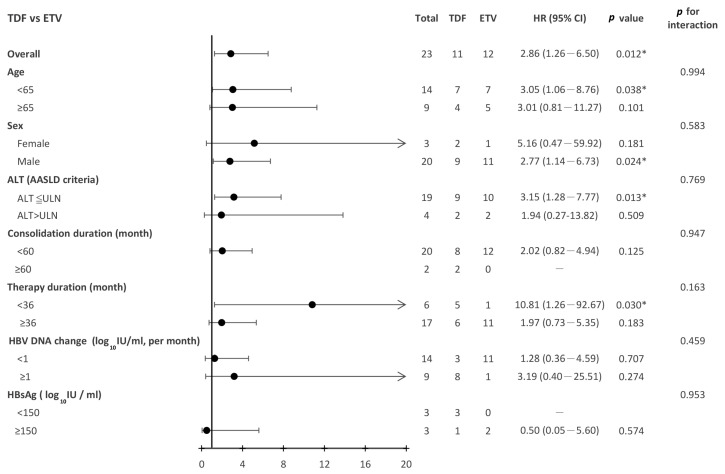
Subgroup analysis for hepatic decompensation for patients discontinuing TDF vs. ETV. TDF, tenofovir disoproxil fumarate. ETV, entecavir. * < 0.05.

The hazard ratios for hepatic decompensation for patients after the discontinuation of TDF versus ETV across the different patient subgroups are shown in Figure 4. The risk of hepatic decompensation after the discontinuation of antiviral therapy was significantly higher in patients discontinuing TDF than in those discontinuing ETV (HR = 2.86; 95%CI 1.26–6.5; *p* = 0.012). The higher risk of hepatic decompensation in patients undergoing the discontinuation of TDF therapy was consistently observed in various subgroups, such as in patients under 65 years of age, those who had undergone previous antiviral therapy less than 36 months, those with an ALT lower than the AASLD criteria threshold, and male patients.

### 3.4. Association between Clinical Outcome and Off-Therapy HBV DNA Change

**Figure 5:**
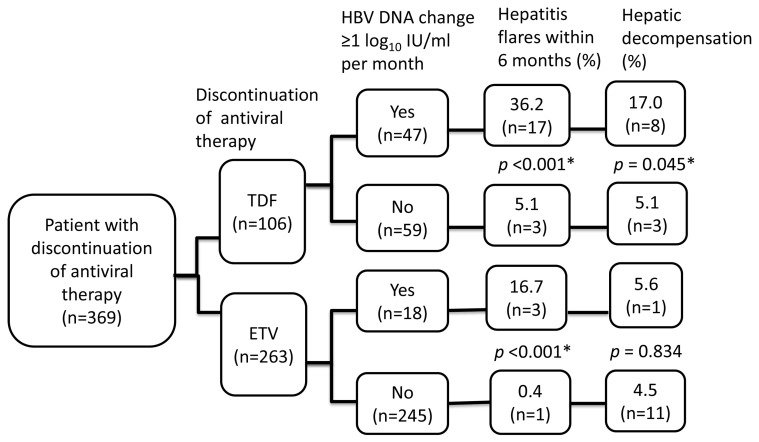
The associations between clinical outcomes and off-therapy HBV DNA changes. * < 0.05.

Figure 5 summarizes clinical outcomes by HBV DNA change after the discontinuation of antiviral therapy. Significantly higher rates of hepatitis flares within 6 months (36.2% vs. 5.1%, *p* < 0.001) and hepatic decompensation (17.0% vs. 5.1%, *p* = 0.045) were observed in patients who had a dynamic change in HBV DNA ≥ 1 log_10_ IU/mL per month after the discontinuation of TDF. Similar findings regarding hepatitis flares were observed in patients who had been treated with ETV (hepatitis flares within 6 months 16.7% vs. 0.4%, *p* < 0.001). Nevertheless, the rates of hepatitis decompensation were similar regardless of the change in HBV DNA after the discontinuation of ETV (5.6% vs. 4.5%, *p* = 0.834). 

## 4. Discussion

In this study, we demonstrated that the discontinuation of TDF and higher levels of ALT with a cut-off value of 35 for men and 25 for women (AASLD criteria threshold) at EOT were associated with an earlier onset of hepatitis flare. The rapid rebound of HBV DNA from the nadir (≥1 log_10_ IU/mL per month) after the discontinuation of antiviral therapy played an important role in the development of earlier hepatitis flares and hepatic decompensation in non-cirrhotic HBeAg-negative CHB patients. Older age was another independent factor for hepatic decompensation after the discontinuation of antiviral therapy.

ETV and TDF, both having high potency and a low resistance rate, are recommended as the first-line antiviral therapy by Clinical Practice Guidelines [7,8,11]. Nevertheless, the guidelines recommend finite therapy in non-cirrhotic HBeAg-negative patients after achieving long-term durable on-therapy viral suppression [7,8]. The off-therapy response pattern may vary according to the prior antiviral therapy. Off-therapy with TDF exhibited the rapid rebound of HBV DNA from the nadir and had a higher rate of clinical relapse than ETV [9,14]. The rapid rebound of HBV DNA after the discontinuation of antiviral therapy is one predictor for earlier-onset and severe hepatitis flares [15]. In our study, the off-therapy dynamic change in HBV DNA (≥1 log_10_ IU/mL per month) and the discontinuation of TDF were predictors of hepatitis flares within 6 months after the discontinuation of antiviral therapy. 

ALT is an indicator of hepatic necroinflammation that can be used to distinguish an inactive carrier from hepatitis [7,16]. The host immune response to HBV virus leads to liver necroinflammation and fibrosis, which contribute to the elevation of ALT [17]. CHB patients with low ALT levels had a low probability of significant liver inflammation and a greater decline in liver stiffness after antiviral therapy [18,19]. Our study showed that patients with ALT lower than the threshold of AASLD criteria at EOT had a lower risk of the early onset of hepatitis flares. This finding might be partly explained by the lower ALT at EOT representing less HBV virus activity and lower-grade fibrosis. Thus, the time from the discontinuation of antiviral therapy to a hepatitis flare may be longer. Further studies are needed to explore this issue.

Hirode G et al. [10] reported that the cumulative incidence of hepatic decompensation after the discontinuation of antiviral therapy among non-cirrhotic HBeAg-negative patients with at least 12 months of consolidation therapy was 0.7%, 0.8%, 0.8%, 1.1%, and 1.1% at 12, 24, 36, 48, and 60 months, respectively. Hsu et al. [20] reported the 4-year cumulative incidence of hepatic decompensation after discontinuing TDF or ETV was 1.1% (95%CI, 0.56–1.97%) in non-cirrhotic HBeAg-negative patients with undetectable HBV DNA at EOT. In our study, the cumulative incidence of off-therapy hepatic decompensation was 2%, 4%, 5%, and 8% at 6, 12, 18, and 24 months, respectively. Such differences may be attributed, at least in part, to the higher proportion of older age of the patients and the lower frequency of off-therapy follow-up in our study. Hsu et al. [20] also reported age (adjusted sub-distribution hazard ratio, 1.21 per 10 years; 95%CI of 1.03–1.42) was a significant risk factor of severe flares with hepatic decompensation after the discontinuation of antiviral therapy, and patients above 50 years of age without cirrhosis or portal hypertension had a higher 4-year cumulative incidence of hepatic decompensation after discontinuing antiviral therapy (1.8% with a 95%CI of 1.3–2.3%). In our study, an off-therapy dynamic change in HBV DNA and older age were associated with hepatic decompensation after discontinuing TDF or ETV. Age has been correlated with the progression of liver fibrosis, and a previous study demonstrated that liver fibrosis increased after the age of 35 and 42 years in HBeAg-positive and HBeAg-negative patients, respectively [21,22]. Evidence suggests that the severity of fibrosis is associated with an increased risk for hepatic decompensation, cirrhosis, hepatocellular carcinoma, and mortality [23,24]. 

There were several limitations to this study. First, this was a retrospective study and the number of cases was relatively small. Therefore, we did not perform propensity score matching to reduce confounding variables at EOT. Second, the qHBsAg level was not available in all patients in this study. Third, hepatitis B core-related antigen (HBcrAg) quantification, a novel serological biomarker that reflects covalently closed circular DNA transcriptional activity [25], was not available in all patients in our study. Huang et al. [26] reported a combination of a pre-treatment qHBcrAg level with a cut-off value of ≤4 log_10_ IU/mL and qHBsAg at EOT with a cut-off value <150 IU/mL can reduce HBV off-therapy relapse in HBeAg-negative non-cirrhotic patients after the discontinuation of ETV. Similar results were observed in those discontinuing TDF. Kuo et al. [27] reported a combination of a pre-treatment qHBcrAg level with a cut-off value of <4.7 log_10_ IU/mL and qHBsAg at EOT with a cut-off value <100 IU/mL can reduce HBV off-therapy relapse in HBeAg-negative non-cirrhotic patients after the discontinuation of TDF. We could not properly elucidate the associations of the pre-treatment qHBcrAg level and qHBsAg at EOT with the risk of off-therapy hepatitis flares within 6 months and hepatic decompensation in our study. Forth, HBV genotype was not determined in this study, and therefore, their effects on off-therapy hepatitis flares within 6 months and hepatic decompensation could not be assessed. Chiu et al. [28] reported that off-therapy virological and clinical relapse occurred more frequently in HBeAg-negative patients with HBV genotype B infection than those with HBV genotype C infection. Fifth, the frequency of follow-up visits and laboratory testing varied. The less frequent off-therapy monitoring of laboratory tests and HBV DNA would delay the detection of hepatic events after the discontinuation of antiviral therapy. Finally, liver biopsies and fibroscans were not performed for all enrolled patients to confirm cirrhosis status prior to previous treatment and at EOT. The assessment of cirrhosis status was based on the characteristic findings of abdominal ultrasonography, which might not have been enough to confirm the diagnosis. 

## 5. Conclusions

This study found that a dynamic change in HBV DNA after the discontinuation of antiviral therapy and off-therapy ALT levels can provide a roadmap for off-therapy surveillance. We could assess the risk of off-therapy HBV relapse by stratifying patients according to off-therapy ALT level (AASLD criteria threshold) initially and then monitor HBV DNA levels regularly, especially in patients discontinuing TDF. If the abrupt elevation of HBV DNA was detected, the risk of hepatitis, and possibly even sequential hepatitis flares or hepatic decompensation, would be elevated. Antiviral therapy should be discontinued cautiously in older-aged patients. This study provides evidence demonstrating the importance of individual off-therapy surveillance. More frequent monitoring is needed in subjects with a greater risk of HBV relapse to reduce the incidence or severity of hepatic events after the discontinuation of antiviral therapy. 

## Figures and Tables

**Table 1:** Baseline characteristics of study population at end of treatment.

	Total (n = 369)	TDF (n = 106)	ETV (n = 263)	*p* Value
Age, years	53.77 (45.14–63.32)	50.60 (41.31–61.42)	54.43 (46.58–64.42)	0.037
Male sex, n (%)	249 (67.30%)	71 (66.98%)	178 (67.68%)	0.897
BMI	24.74 (21.89–26.89)	24.77 (20.63–27.74)	24.72 (22.17–26.72)	0.871
AFP, ng/mL	3.03 (2.295–4.5225)	3.03 (2.34–4.55)	3.02 (2.25–4.5)	0.574
PLT, 10^3^/μL	203 (156.5–238.5)	205.5 (181–257)	197 (153–227)	0.060
ALT, U/L	25 (18–38)	27 (19.5–41.75)	25 (17–35)	0.071
APRI *	0.31 (0.22–0.43)	0.31 (0.23–0.44)	0.3 (0.22–0.41)	0.805
FIB-4 **	1.32 (0.94–2.05)	1.14 (0.85–1.99)	1.36 (0.98–2.08)	0.121
PT, s	10.3 (10.1–10.7)	10.5 (10.25–10.7)	10.2 (10–10.7)	0.168
INR	1.01 (0.9875–1.05)	1.01 (1.01–1.06)	1.01 (0.98–1.05)	0.474
Cr, mg/dL	0.93 (0.77–1.09)	0.93 (0.83–1.08)	0.92 (0.76–1.09)	0.532
eGFR, mL/min/1.73 m^2^	84.63 (72–97.295)	85 (72–98)	84 (71.5–97)	0.753
Consolidation duration, months	30.65 (28–33)	30.67 (29.26–33.36)	30.65 (27.73–33.02)	0.599
Therapy duration, months	36 (36–37)	36.01 (35.98–38.03)	36.01 (36.01–37.13)	0.658
HBsAg, log_10_ IU/mL	2.4 (1.8–2.9)	2.56 (1.84–3)	2.36 (1.76–2.83)	0.097

Abbreviation: TDF, tenofovir disoproxil fumarate; ETV, entecavir; BMI, Body Mass Index; AFP, Alpha-fetoprotein; PLT, platelet; ALT, alanine aminotransferase; APRI, aspartate aminotransferase to platelet ratio index; FIB-4, Fibrosis (FIB)-4; PT, prothrombin time; INR, international normalized ratio; Cr, creatinine; eGFR, estimated glomerular filtration rate. * APRI = (AST [U/L] × 100)/(ULN_AST_ × platelet count [10^9^/L]) ** FIB-4 = Age (years) × AST (U/L)/platelet count (×10^9^/L) × √ALT (U/L). Mann–Whitney test. Chi-Square test. Continued variables were presented as median ± interquartile range (IQR); categorical variables are indicated as numbers of cases and percentages.

**Table 2:** Factor with hepatitis flares within 6 months in patients after discontinuing TDF or ETV.

	Univariate Model			Multivariate Model		
	HR	(95%CI)	*p* Value	HR	(95%CI)	*p* Value
Age, years	1.00	(0.97–1.03)	0.930	1.02	(0.98–1.06)	0.269
Male, sex	1.18	(0.49–2.84)	0.715	1.44	(0.49–4.23)	0.503
BMI	0.99	(0.85–1.16)	0.905			
AFP, ng/mL	1.04	(0.94–1.16)	0.431			
PLT, 10^3^/μL	1.00	(0.93–1.01)	0.685			
ALT, U/L	1.01	(1.00–1.02)	0.170			
ALT ≤ ULN *	Reference			Reference		
ALT > ULN *	2.48	(1.05–5.85)	0.038			
ALT ≤ ULN (AASLD criteria) **	Reference			Reference		
ALT > ULN (AASLD criteria) **	5.58	(2.20–14.15)	<0.001	4.93	(1.84–13.18)	0.001
APRI ***	1.53	(0.37–6.40)	0.560			
FIB-4 ****	1.14	(0.79–1.66)	0.477			
PT, s	0.08	(0.00–1.30)	0.075			
Cr, mg/dL	0.97	(0.51–1.83)	0.913			
eGFR, mL/min/1.73 m^2^	1.01	(0.98–1.03)	0.520			
HBV DNA change ≥ 1 log_10_ IU/mL per month	29.95	(10.21–87.84)	<0.001	10.70	(3.30–34.68)	<0.001
Consolidation duration, months	0.98	(0.95–1.02)	0.429			
Therapy duration, months	1.01	(0.99–1.03)	0.231			
HBsAg, log_10_ IU/mL	1.28	(0.76–2.15)	0.347			
TDF vs. ETV	13.88	(4.74–40.61)	<0.001	6.44	(1.70–24.01)	0.006

Abbreviation: BMI, Body Mass Index; AFP, Alpha-fetoprotein; PLT, platelet; ALT, alanine aminotransferase; APRI, aspartate aminotransferase to platelet ratio index; FIB-4, Fibrosis (FIB)-4; PT, prothrombin time; Cr, creatinine; eGFR, estimated glomerular filtration rate; TDF, tenofovir disoproxil fumarate; ETV, entecavir. * ALT ULN (upper limit of normal range) was set at 50 U/L for men and 35 U/L for women. ** normal ALT according to AASLD criteria was set at 35 U/L for men and 25 U/L for women. *** APRI = (AST [U/L] × 100)/(ULN_AST_ × platelet count [10^9^/L]). **** FIB-4 = Age (years) × AST (U/L)/platelet count (×10^9^/L) × √ALT (U/L).

**Table 3:** Factor with hepatic decompensation in patient after discontinuing TDF or ETV.

	Univariate Model			Multivariate Model		
	HR	(95%CI)	*p* Value	HR	(95%CI)	*p* Value
Age, years	1.05	(1.02–1.09)	0.001	1.06	(1.03–1.10)	<0.001
Male sex	3.27	(0.97–10.99)	0.056	3.40	(1.00–11.52)	0.050
BMI	1.04	(0.91–1.19)	0.559			
AFP, ng/mL	0.94	(0.72–1.23)	0.670			
PLT, 10^3^/μL	0.99	(0.99–1.01)	0.777			
ALT, U/L	1.00	(0.99–1.02)	0.663			
ALT ≤ ULN *	Reference			Reference		
ALT > ULN *	1.57	(0.58–4.26)	0.378			
ALT ≤ ULN(AASLD criteria) **	Reference			Reference		
ALT > ULN (AASLD criteria) **	1.78	(0.77–4.12)	0.179			
APRI ***	1.92	(0.49–7.52)	0.352			
FIB-4 ****	1.25	(0.94–1.65)	0.122			
PT, s	1.39	(0.47–4.08)	0.552			
Cr, mg/dL	1.06	(0.70–1.60)	0.785			
eGFR, mL/min/1.73 m^2^	0.98	(0.96–1.002)	0.078			
HBV DNA change ≥ 1 log_10_ IU/mL per month	5.01	(2.15–11.65)	<0.001	3.26	(1.14–9.33)	0.028
Consolidation duration, months	0.99	(0.96–1.03)	0.994			
Therapy duration, months	1.00	(0.97–1.03)	0.936			
HBsAg, log_10_ IU/mL	0.82	(0.47–1.46)	0.507			
TDF vs. ETV	2.86	(1.26–6.50)	0.001	2.03	(0.73–5.65)	0.176

Abbreviation: BMI, Body Mass Index; AFP, Alpha-fetoprotein; PLT, platelet; ALT, alanine aminotransferase; APRI, aspartate aminotransferase to platelet ratio index; FIB-4, Fibrosis (FIB)-4; PT, prothrombin time; Cr, creatinine; eGFR, estimated glomerular filtration rate; TDF, tenofovir disoproxil fumarate; ETV, entecavir. * ALT ULN (upper limit of normal range) was set at 50 U/L for men and 35 U/L for women. ** normal ALT according to AASLD criteria was set at 35 U/L for men and 25 U/L for women. *** APRI = (AST [U/L] × 100)/(ULN_AST_ × platelet count [10^9^/L]). **** FIB-4 = Age (years) × AST (U/L)/platelet count (×10^9^/L) × √ALT (U/L).

## Data Availability

The data presented in this study are available on request from the corresponding author. The data are not publicly available due to privacy/ethical restrictions.
